# Digital Histopathological Discrimination of Label-Free Tumoral Tissues by Artificial Intelligence Phase-Imaging Microscopy

**DOI:** 10.3390/s22239295

**Published:** 2022-11-29

**Authors:** José Luis Ganoza-Quintana, José Luis Arce-Diego, Félix Fanjul-Vélez

**Affiliations:** Biomedical Engineering Group, TEISA Department, Universidad de Cantabria, Av. de los Castros 46, 39005 Santander, Spain

**Keywords:** digital histology, tumor discrimination, biomedical optical microscopy, phase-imaging, machine learning, artificial intelligence

## Abstract

Histopathology is the gold standard for disease diagnosis. The use of digital histology on fresh samples can reduce processing time and potential image artifacts, as label-free samples do not need to be fixed nor stained. This fact allows for a faster diagnosis, increasing the speed of the process and the impact on patient prognosis. This work proposes, implements, and validates a novel digital diagnosis procedure of fresh label-free histological samples. The procedure is based on advanced phase-imaging microscopy parameters and artificial intelligence. Fresh human histological samples of healthy and tumoral liver, kidney, ganglion, testicle and brain were collected and imaged with phase-imaging microscopy. Advanced phase parameters were calculated from the images. The statistical significance of each parameter for each tissue type was evaluated at different magnifications of 10×, 20× and 40×. Several classification algorithms based on artificial intelligence were applied and evaluated. Artificial Neural Network and Decision Tree approaches provided the best general sensibility and specificity results, with values over 90% for the majority of biological tissues at some magnifications. These results show the potential to provide a label-free automatic significant diagnosis of fresh histological samples with advanced parameters of phase-imaging microscopy. This approach can complement the present clinical procedures.

## 1. Introduction

The gold standard of present clinical diagnosis is based on histopathology [1,2]. Conventional biopsy implies physical extraction of the tissue sample, fixation, inclusion, staining, and finally observation by an optical microscope. This process is time-consuming, entails manipulation complexity, and remains pathologist-dependent. The artifacts that can arise from the sample manipulation and the pathologist bias, reduce the diagnosis accuracy [3]. It is possible to improve the outcome of the histopathological diagnostic procedure by digital histology [4]. In digital histology, optical microscopy images of the histological samples are digitalized. This fact allows for semiautomatic or automatic image processing of conventional intensity microscopy images. Usual image processing techniques employed on these images are de-noising, pattern recognition, edges identification, or color enhancement, among others. If the sample presents markers, usually fluorophores, then digital histology contributes to a better location of the emission sites. These sites are correlated with otherwise low-contrast areas, such as cellular nuclei or tumoral tissue [5,6]. Digital histology can also provide a 3D reconstruction of the biological sample from the 2D images of the tissue slices. This 3D image makes it easier for the pathologist to identify the real spatial structure of the original sample [7]. Measurements of specific tissue structures with diagnostic significance can be also made [8]. Digital histology can provide quantitative parameters that contribute to semiautomatic or even automatic diagnosis [4]. Although digital histology can improve histopathological diagnosis, it usually presents an intrinsic limitation. It is based on the same intensity microscopy images that the pathologists employ in the conventional procedure. This fact implies that the sample processing remains unchanged, so the procedure is still time-consuming and with potential artifacts.

The diagnostic procedure would greatly benefit from a label-free diagnosis. Label-free diagnosis employs biological samples without further manipulation. According to the previously described histopathological diagnostic procedure, the sample would not be fixated, included, nor stained [2]. This approach presents the effect of dramatically reducing time to diagnosis, and prevents processing artifacts from appearing in the images. On the other hand, the intrinsic contrast of biological tissues, particularly of thin slices that are highly transparent, is usually quite low [9]. Low contrast greatly complicates the identification of areas of interest in the samples and, as a consequence, tissue diagnosis. Optical radiation-tissue interaction can be increased by using thicker samples [10]. However, in this case the effect of optical scattering increases, and this fact limits image resolution and depth in the tissue [10]. This effect can be partially overcome by optical tomographic techniques, such as Optical Coherence Tomography [11]. The problem is that image resolution is around 1 micron, and histopathology is greatly based on optical microscopy high resolution that is diffraction-limited, or even below [12]. Some techniques for increasing contrast in thin biological tissue slices have been proposed, either extrinsic, mainly fluorescence based [13], or intrinsic for label-free diagnosis, based on spectroscopy [14] or polarimetry [15,16]. These approaches are part of the so-called optical biopsy that tries to overcome the drawbacks of conventional biopsy with optical techniques [17]. However, until now, no optical technique has been able to provide better diagnostic results than conventional histology.

Low contrast thin biological tissues are usually observed by phase-contrast microscopy (PCM), typical for cell cultures [18]. This technique exploits the variation of refractive index in different parts of the biological tissues. This variation generates light scattering that can interfere with a reference signal due to phase differences, in a procedure similar to holography [19]. The usual diagnostic use of PCM is based on qualitative visual identification of structures. Phase differences in biological tissues can be made quantitative by advanced microscopy setups [20], such as Spatial Light Interference Microscopy (SLIM) [21], or Digital Holographic Microscopy (DHM) [22]. These techniques provide highly accurate quantitative phase information, usually with more complex setups [23], and even including color information on the images [24]. This diagnostic information has proven to be significant preferential in cells and, in some cases, in conventional histological tissues [25]. The theory of light propagation in turbid media has been also applied to biological tissues [26], but the complexity of propagation makes it difficult to find useful diagnostic parameters. Several parameters that can be extracted from phase-contrast images have been proposed, such as the scattering coefficient [27] or the refractive index variance. These parameters have been applied mainly to isolated cells, or to conventional fixed histological tissues with the advanced quantitative setups previously mentioned. These advanced setups, for example SLIM or DHM, are able to provide accurate high-resolution phase information, usually at an intracellular level [21]. However, they are usually complex and expensive and need specific staff training. For these reasons, these devices are difficult to find at a histopathology service of a general hospital. The interest of histopathology services is mainly focused on the whole sample, in order to provide a positive or negative diagnosis, and not necessarily at the cellular level. On the contrary to these complex devices, phase-contrast microscopy is commonly available at these clinical services. The implementation of advanced diagnostic label-free approaches would be much more feasible at histopathology services if the phase-contrast parameters extracted from phase-contrast images would have diagnostic potentiality. To this aim, several phase-contrast parameters have been previously evaluated as potential intrinsic biomarkers on fresh histological samples of kidney, colon, and liver [28]. The results show promising statistical potential in the discrimination of healthy and tumoral samples. However, no automatic classification procedures based on artificial intelligence were applied, and the results were limited to three biological tissue types.

The application of artificial intelligence, mainly deep learning, to digital histology is greatly improving the diagnostic outcome [29]. Present approaches are based on conventional fixed histological images that are first digitalized [30]. The application of deep learning by neural networks has been demonstrated on whole slide images of conventional histological samples for breast tumor detection [30,31], gastric and colonic tumors [32], or prostate cancer [33]. The deep learning algorithms can then take advantage of the additional contrast that staining introduces in the histological samples, including color information, and other features that are sometimes hand-crafted [30]. The dependence on the exact way the samples have been processed influences the classification results, particularly when samples come from different services [34]. Most of the algorithms are more or less complex convolutional networks based on a great variety of parameters, and they are usually applied to the whole image on a pixel-basis. This makes the classifier more difficult to train (something that should be made at the service due to the dependence on processing protocols), and the classification process slower. These characteristics present difficulties in the assumption of the technique by histopathology services. On the contrary, the use of classification algorithms that are easier to train and implement and faster to get a result, such as linear discriminants, k-nearest neighbors, or support vector machines, could facilitate adoption if successful. Employing phase-contrast parameters from label-free histological slides reduces the dimensionality of the input data and further contributes to this aim. Label-free fresh histological samples reduce contrast in the images, but they make the analysis more service-independent, as several processing steps (fixation and staining) are not implemented, what also makes time to diagnosis shorter.

In this work, we propose, implement and evaluate classification systems based on artificial intelligence for the automatic or semiautomatic healthy and tumoral tissue discrimination based on fresh histological samples. Phase-contrast microscopy images of fresh histological samples at three different magnifications, 10×, 20× and 40× were obtained. Samples of liver, kidney, ganglion, testicle, and brain were employed. Advanced phase-contrast parameters, such as refractive index variance, scattering coefficient, anisotropy of scattering, fractal dimension and outer scale were calculated from the images. The potentiality of discrimination of each parameter for each biological tissue type and magnification was first evaluated by a statistical ANOVA approach. Several classification algorithms are applied to the previously identified parameters, such as linear (LDA) and quadratic (QDA) discriminant analysis, Naïve Bayes (NB) and kernel Naïve Bayes (kNB), k-nearest neighbors (kNN), support vector machine (SVM), decision tree (DT), and artificial neural network (ANN). The results of the classification for each biological tissue type, magnification and classifier were analyzed. The potential clinical applicability of the results was evaluated, based on the classification error metrics.

## 2. Materials and Methods

The materials and methods involved in this article are phase-contrast microscopy setup, advanced phase-contrast parameters definition calculation, fresh histological samples, statistical analysis, and classification algorithms. Each of these elements is described in the following subsections.

### 2.1. Phase-Contrast Microscopy

**Figure 1:**
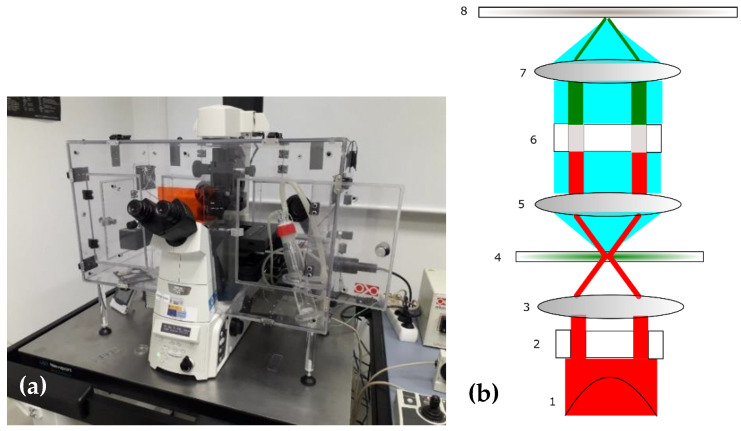
(**a**) Phase-contrast microscope employed for imaging the fresh samples; (**b**) Schematic representation of the phase-contrast microscope, with the non-scattered (in red) and scattered (in blue) optical fields: 1, optical source; 2, condenser annulus; 3, condenser; 4, sample; 5: collimating lens; 6, phase and attenuation filter; 7: imaging lens; 8: image plane.

Phase-contrast microscopy images were obtained from fresh histological samples by an advanced setup, including motorized sample movement [28]. Samples were placed on the motorized stage, and phase-contrast was produced by a combination of a phase ring and specific objectives. The objectives used for the different magnifications were Nikon 10× Plan Fluor 0.30NA Ph1 DLL, 20× SPlan Fluor ELWD 0.45 NA ph1 ADM, and 40× SPlan Fluor ELWD 0.60 NA ph2 ADM. The motorized stage gave the possibility to automatically obtain large images composed of several microscope fields. Images were recorded by a 12-bit gray-scale CCD camera (Orca-R2, Hamamatsu). Samples are scanned at several locations, avoiding fields in which pure glass slide was dominant against biological tissue. Figure 1a shows the phase-contrast microscope, and a schematic representation of the phase-contrast microscopy main elements is included in Figure 1b.

### 2.2. Fresh Histological Samples

Biological samples were obtained from a specific biobank belonging to the Marqués de Valdecilla University Hospital (Santander, Spain). The biobank is focused on the collection and maintenance of different biological samples of patients that underwent diagnostic procedures or make donations of samples. The aim of the samples stored in the biobank is to employ the collected samples in research activities. Informed consent of the patients was asked before including each sample in the biobank, following biobank ethical guidelines.

Fresh histological samples of less than 6 μm were sliced by a cryo-microtome from frozen biological tissues. No conventional histological procedures were applied, such as fixation, inclusion or staining. Samples were positioned on treated microscope slides to maximize adherence and remain frozen. Samples were located at 4 °C three hours before starting measurements. Healthy and tumoral biological tissues of liver, kidney, ganglion, testicle and brain were extracted, with a total of 37 samples. 

### 2.3. Advanced Phase-Contrast Parameters

Phase-contrast parameters were derived from the refractive index changes in the biological tissue, which are associated to the intensity variations of the phase-contrast image [20,25,28]. Refractive index variation can be considered as a random process, in which there is no temporal variation of the samples. In this sense, the samples are turbid according to light propagation [26]. The first moment of the refractive index of the sample is its mean $n_{0}$. As the sample thickness is low, it can be assumed that the sample is a weakly scattering medium. In this case, the dispersion relation can be written as:

(1) $$\langle k^{2}\rangle =n_{0}^{2}\beta_{0}^{2}\left(1+\frac{\sigma_{n}^{2}}{n_{0}^{2}}\right)$$

In this equation, $\langle k^{2}\rangle$ is the second order moment of the wavevector k, $\beta_{0}=\omega /c$ is the wavenumber in vacuum, and $\sigma_{n}^{2}$ is the refractive index spatial variance. From this expression it is possible to find an equation that relates phase variation with refractive index variance [25], in terms of the mean gradient intensity of the phase-contrast image ∇ϕ:

(2) $$\sigma_{n}^{2}=\frac{1}{\beta_{0}^{2}}\langle {\left|\nabla \phi \right|}^{2}\rangle$$

The refractive index variance will be the first phase-contrast parameter to be employed in the system. As the phase-contrast is based on an interferogram built with the scattered by the sample and non-scattered optical signals, scattering parameters can also be estimated from phase-contrast images [27]. The scattering-phase theorem states that the scattering coefficient $\mu_{s}$ can be estimated from the phase spatial variance ${\langle \Delta \emptyset^{2}\left(r\right)\rangle }_{r}$ and the sample thickness L:

(3) $$\mu_{s}=\frac{{\langle \Delta \emptyset^{2}\left(r\right)\rangle }_{r}}{L}$$

The same theorem allows to calculate the anisotropy of scattering g. This parameter is quite relevant in biological tissues, as the usual structural sizes of internal heterogeneities give values near 1, indicating strongly forward scattering. The expression as a function of the incident wavevector $k_{0}$ is:

(4) $$g=1-\frac{1}{2k_{0}^{2}}\frac{{\langle {\left|\nabla \left[\emptyset \left(r\right)\right]\right|}^{2}\rangle }_{r}}{{\langle \Delta \emptyset^{2}\left(r\right)\rangle }_{r}^{2}},$$

These three parameters describe the refractive index variability and scattering properties of the sample, extracted from the phase-contrast images. Another parameter of interest can be calculated from the theory of turbulent media [26]. Although, as previously stated, this theory comprises general media with temporal variations of the properties, it can be applied to turbid tissue as well. Media under this model present parameters called turbulent eddies. Eddies in the context of phase-contrast images are irregularities in the refractive index. According to the particular medium, the largest possible eddy is called the outer scale of turbulence $L_{0}$, and the smallest is the inner scale. The outer scale can be obtained from the spectrum of refractive index heterogeneities. In the case of biological tissues, this spectrum can be usually approximated by a Von Kármán expression with an exponent m [35]:

(5) $$\Phi \left(\kappa \right)=\frac{4\pi \sigma_{n}^{2}L_{0}^{2}\left(m-1\right)}{{\left(1+\kappa^{2}L_{0}^{2}\right)}^{m}}$$

Finally, the fractal dimension is another parameter of potential interest. It can be estimated from the exponent m of the previous expression by $d_{f}=4-m$, or it can be calculated by different numerical approaches, such as box-counting, correlation, sandbox, or Fourier spectrum [36]. 

The proposed phase-contrast parameters are strongly related with refractive index distribution in the tissue (refractive index variation, scattering coefficient, anisotropy of scattering, fractal dimension or outer scale). As they are calculated for the whole microscope image, a reasonable estimation of this distribution is expected from phase-contrast images. All these parameters have shown already statistical potential for tumoral tissue discrimination as stated previously [28].

### 2.4. Statistical Analysis

Mean and standard deviation were calculated for the measurements of each parameter made on each biological tissue type, distinguishing healthy or tumoral state, and magnification. The quantification of the potential capability of each parameter was made quantitatively by an ANOVA analysis. The statistical ANOVA analysis tests the hypotheses of the average values of several variables of interest being equal or not. In our case, we have two groups, healthy and tumoral, for each tissue type that we would like to distinguish. ANOVA analysis will calculate the Snedecor F and the *p*-value for each case. If the Snedecor F is high, or equivalently if the *p*-value is sufficiently low, then the hypothesis of the average values being equal is false. This result is an indication of differentiation in the average values of both cases, and as a consequence a higher potentiality to be a discrimination parameter. A *p*-value below 0.005, or 95% confidence, is usually employed as a reference for statistical significance.

### 2.5. Classification Algorithms

Several classification algorithms were employed to implement and evaluate the automatic diagnostic capabilities of the previous parameters. These algorithms are based on artificial intelligence and machine learning [37,38]. Similar approaches have been previously applied to other classification problems for diagnosis, for instance in diffuse reflectance spectroscopy [39]. In this work, generative algorithms, such as linear (LDA) and quadratic (QDA) discriminant analysis, and normal (NB) and kernel Naïve-Bayes (kNB), as long as discriminative algorithms, such as k-nearest neighbors (kNN), decision tree (DT), artificial neuronal networks (ANN) or support vector machines (SVM), are implemented. LDA and QDA employ a null threshold for the linear coefficient, and prior probabilities defined as the relative frequencies. kNB uses a kernel smoothing density estimate. kNN is based on the Euclidian distance with a number of neighbors *k* = 5. SVM employs a multi-class error-correcting output codes model based on binary support vector machine by a one-versus-one coding design. DT is based on binary classification, with a minimum size of parent nodes equal to 10. ANN is implemented by 10 hidden layers, trained with a scaled conjugate gradient back propagation function, and a cross-entropy loss function.

The evaluation of the classifiers was made by means of mainly two parameters: re-substitution and cross validation errors. The re-substitution error was calculated by first training the classifier with all available data, and afterwards employing the same data as input to be classified by the system. This approach, although interesting for a first evaluation of the classifier, is not realistic, as in the final application samples that were not used for training will have to be classified. For this reason, the cross-validation error, that employs a dataset for training the classifier and a different dataset for testing, was also calculated. This process was repeated for several combinations of training and testing datasets to prevent any bias in the selection of groups.

## 3. Results

### 3.1. Phase-Contrast Microscopy Images

**Figure 2:**
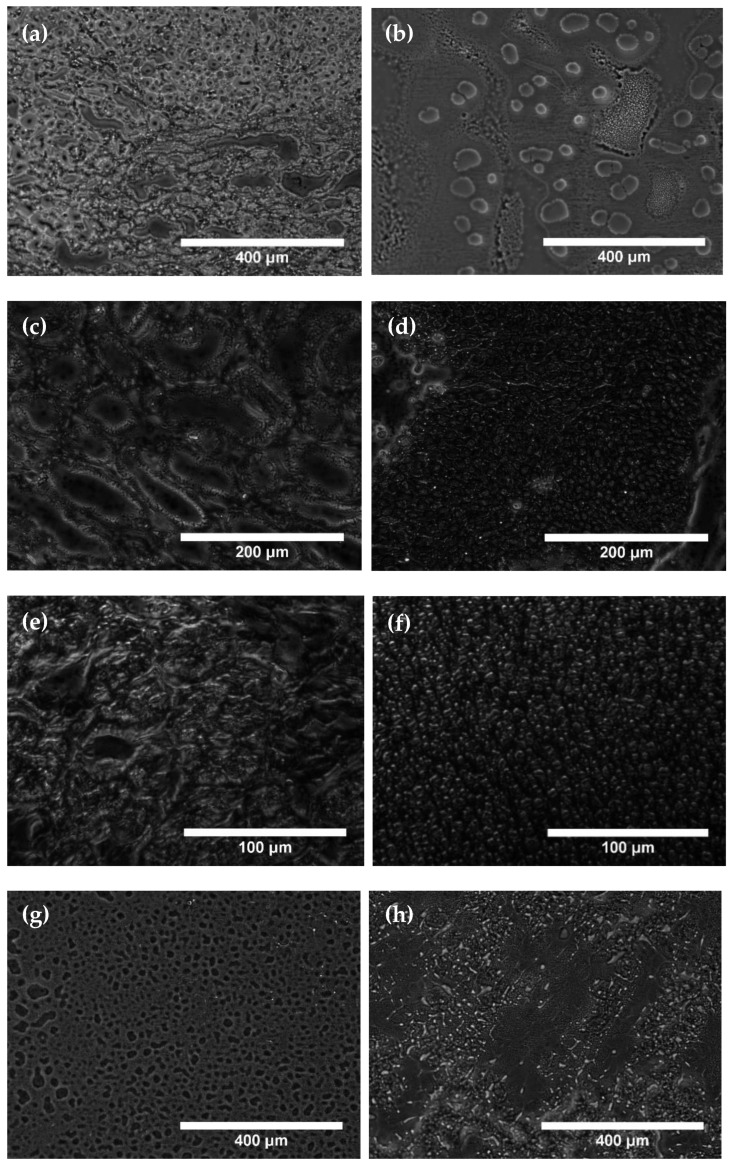

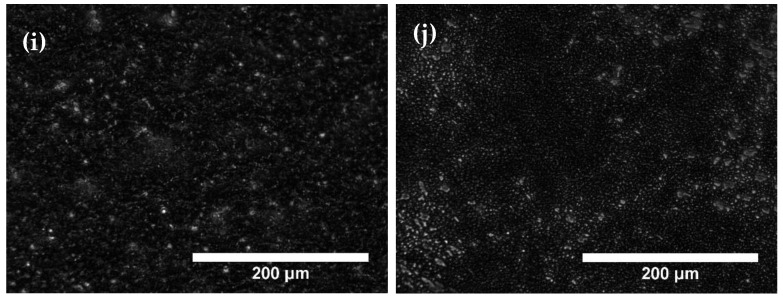
Phase-contrast images examples of the different tissue types, state and magnification: (**a**) Healthy liver at 10×; (**b**) Tumoral liver at 10×; (**c**) Healthy kidney at 20×; (**d**) Tumoral kidney at 20×; (**e**) Healthy ganglion at 40×; (**f**) Tumoral ganglion at 40×; (**g**) Healthy testicle at 10×; (**h**) Tumoral testicle at 10×; (**i**) Healthy brain at 20×; (**j**) Tumoral brain at 20×.

Phase-contrast microscopy images were obtained from the fresh histological samples. A total of 1734 images are obtained (516 for liver, 516 for kidney, 324 for ganglion, 216 for testicle, and 162 for brain). The unequal number of images is due mainly to the different size of each sample on the microscope slide, motivated by the original size of the biological tissue from which the sample was extracted. Microscopic images in which tissue was not present in the vast majority of the field of view were discarded, in order to avoid classification alterations by empty areas. Three different magnifications are used, with the following scales: 866 μm × 660 μm, pixel size 0.64 μm for 10x; 433 μm × 330 μm, pixel size 0.3225 μm for 20x; and 219 μm × 167 μm, pixel size 0.1632 μm for 40x. Some images of each tissue type appear in Figure 2.

As it can be seen in the images of Figure 2, which includes examples of the pairs of images that showed more visual differences, these differences between healthy and tumoral tissues are not generally obvious from the direct visual inspection of the images. Phase-contrast parameters were calculated from the whole pool of images in the next section. 

### 3.2. Phase-Contrast Parameters

Phase-contrast parameters were calculated for each of the images, according to the procedures described in the previous sections. Average and standard deviation values were calculated for each tissue type, state and magnification. The results appear in Table 1.

As it can be appreciated in Table 1, the average values of healthy and tumoral tissues present differences for some parameters and magnifications, compared with the standard deviations, while others are quite similar. A first, a statistical analysis is made in the next section to quantify the potential significance of these differences for classification.

### 3.3. ANOVA Statistical Analysis

The previous results of phase-contrast parameters are analyzed by an ANOVA test. The results appear in Table 2.

Table 2 shows the results of the *p*-value ANOVA test, for each tissue type, magnification and parameter. As said before, lower values of the *p*-value indicate a statistically significant difference in the average values of the healthy and tumoral samples. This is related with the expected classification capability of each phase-contrast parameter and magnification for each tissue type. The next section shows the results of the implementation of different automatic classifiers.

### 3.4. Classification Results

The previously exposed classification algorithms were implemented and applied to the phase-contrast parameters. All the phase-contrast parameters were employed in each classifier to improve performance. The aim was to distinguish between healthy and tumoral samples, for each tissue type and magnification. The capabilities of the classifiers were evaluated with the re-substitution and cross-validation errors. In each of these errors, the results distinguish between the false positive and false negative rates. This distinction allows to consider sensibility and specificity. The results of the re-substitution errors for each tissue type, magnification and classification algorithm are shown in Table 3.

**Figure 3:**
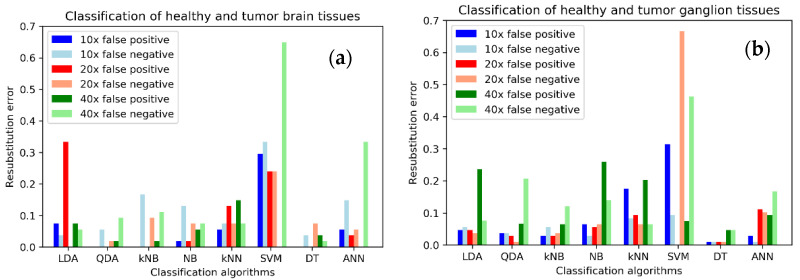

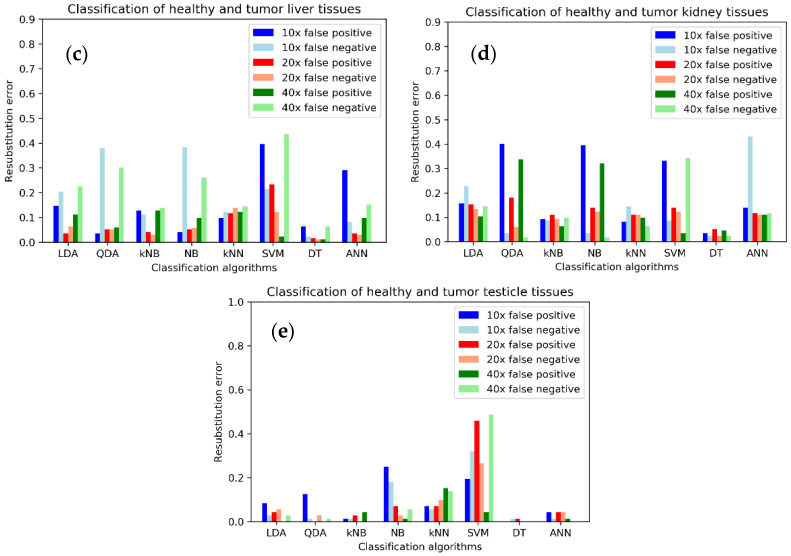
Re-substitution error, distinguishing the false positive and false negative rates, for each tissue type, classifier and magnification: (**a**) Brain tissue; (**b**) Ganglion tissue; (**c**) Liver tissue; (**d**) Kidney tissue; (**e**) Testicle tissue. LDA: linear discriminant analysis; QDA: quadratic discriminant analysis; NB: Naïve Bayes; kNB: kernel Naïve Bayes; kNN: k-nearest neighbors; SVM: support vector machine; DT: decision tree; ANN: artificial neural network.

The information on Table 3 can be better analyzed graphically. Figure 3 shows the re-substitution errors for each of the tissue types.

The results in Table 3 and Figure 3 show a great heterogeneity between the different classifiers, tissue types and magnifications, in terms of the re-substitution error. The cross-validation error is also calculated for all the cases. The results appear in Table 4.

**Figure 4:**
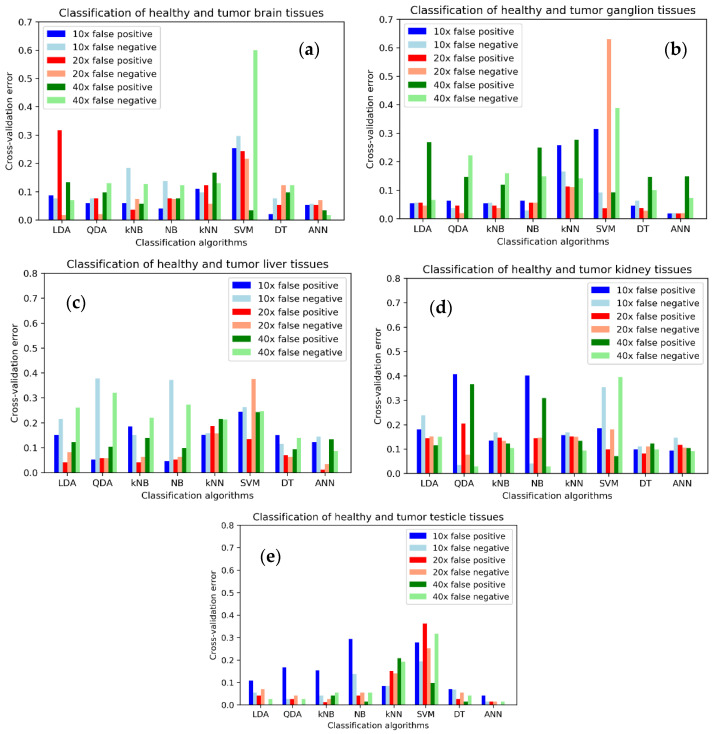
Cross-validation error, distinguishing the false positive and false negative rates, for each tissue type, classifier and magnification: (**a**) Brain tissue; (**b**) Ganglion tissue; (**c**) Liver tissue; (**d**) Kidney tissue; (**e**) Testicle tissue. LDA: linear discriminant analysis; QDA: quadratic discriminant analysis; NB: Naïve Bayes; kNB: kernel Naïve Bayes; kNN: k-nearest neighbors; SVM: support vector machine; DT: decision tree; ANN: artificial neural network.

Similarly, the information of Table 4 is expressed graphically in Figure 4.

Cross-validation errors are, in general, bigger than re-substitution errors, as expected. There is a great variability depending on the tissue type, classifier, and magnification, as it was the case for the re-substitution error.

## 4. Discussion

The proposed system for the automatic discrimination of healthy and tumoral tissues is based on phase-contrast parameters, calculated from phase-contrast images of fresh biological tissue slices, in this case of 6 µm thickness, although the system could be trained for other thicknesses. Examples of phase-contrast images are presented in Figure 2. Images with 10× (Figure 2a,b,g,h), 20× (Figure 2c,d,i,j) and 40× (Figure 2e,f) magnifications are shown, both for healthy (Figure 2a,c,e,g,i) and tumoral (Figure 2b,d,f,h,j) tissues. From direct visual inspection, the differences between healthy and tumoral samples are not evident in general, as can be seen, for instance, by comparing Figure 2i,j. There is an influence of the particular structure inside the tissue slice that is part of the field of view, as it can be appreciated for instance when comparing Figure 2a,b, where the latter shows quasi-elliptical structures that are more difficult to see in the former. However, the presence of these structures does not seem to be distinctive for tumoral tissue as in Figure 2b. Comparing Figure 2c,d, the former corresponding to healthy tissue presents ovoidal structures that are hardly seen in the tumoral tissue in Figure 2d. Regarding magnification, 40× images, Figure 2e,f, do not show more distinction in direct view than images at 10×, Figure 2a,b,g,h, or than images at 20×, Figure 2c,d,i,j. Consequently, it is difficult to provide a diagnosis based on the direct observation of the images. For this reason, and also due to the convenience of automatic digital diagnosis [4], phase-contrast parameters were calculated from the phase-contrast images.

The results of phase-contrast parameters calculations of all the images obtained are shown in Table 1. As described in the previous sections, the considered phase-contrast parameters are the refractive index variance, the scattering coefficient, the anisotropy of scattering, the fractal dimension and the outer scale. Table 1 shows the average values, as well as the standard deviations, of every tissue type (liver, kidney, ganglion, testicle and brain), state (healthy H or tumoral T) and magnification (10×, 20×, and 40×). The discrimination capability of each parameter for each tissue type and magnification is dependent on the difference between the average of healthy and tumoral samples of that tissue type and magnification, and its comparison with the standard deviations. If this difference of the average values of a particular parameter is big compared with standard deviations of the sample, then it is likely that a calculation of the parameter of a sample at one tissue state, either healthy or tumoral, can be easily distinguished from a sample at the other pathological state. If not, then it would be more complex to discriminate based on that parameter at a particular magnification. For instance, Table 1 shows that the scattering coefficient (SC) of ganglion at 20× is 43.46 ± 12.91 for healthy samples, and 18.07 ± 15.31 for tumoral samples. The difference between the average values is 25.39, that is greater than the standard deviations, 12.91 and 15.31. As said, this fact is indicative of the potentiality of scattering coefficient at 20× to be a good discriminator for ganglion tissues. On the other hand, the same parameter for ganglion at 10× is, according to Table 1, 11.10 ± 15.23 for healthy tissue and 16.37 ± 26.96 for tumoral samples. The difference between the average values is 5.27, that is smaller than the standard deviations, 15.23 and 26.96. The results at 40× for the ganglion are 18.39 ± 11.12 for healthy samples, and 22.74 ± 16.10 for tumoral samples, with a difference of 4.35, again much smaller than 11.12 and 22.74, the standard deviations. As these two last cases demonstrate, the scattering coefficient at 10× and 40× for ganglion is not a potentially appropriate parameter to make a significant discrimination of healthy and tumoral tissues. The case of the ganglion demonstrates a significant dependence on the magnification for the discriminative potential of a phase-contrast parameter. Refractive index variance (RIV) of the same tissue, ganglion, according again to Table 1, shows differences in average values of 0.0067, 0.0062 and 0.0018, and standard deviations of 0.0042 and 0.0043, 0.0021, and 0.0063, and 0.0042 and 0.0035, at 10×, 20×, and 40×, respectively. Following the same analysis as before, there is potentiality of discrimination at 10×, as the difference of the averages, 0.0067, is bigger than the standard deviations, 0.0042 and 0.0043. However, this potentiality is severely reduced at 20×, with difference in the average values of 0.0062 and standard deviations of 0.0021 and 0.0063, where the second standard deviation is similar to the difference. At 40×, the difference in average values 0.0018 is much smaller than the standard deviations, 0.0042 and 0.0035, and as a consequence the discriminative potential is compromised. These results show that, for the ganglion, the refractive index variance could be significant at 10×, but not at 20× or 40×. The scattering coefficient previously analyzed showed significance at 20×, but not at 10× or 40×. Consequently, the discrimination capability depends also on the particular phase-contrast parameter, as expected, and its combination with the magnification factor. A tissue type dependence can also be easily shown from Table 1. For the case of the testicle, the refractive index variance presents a difference of averages of 0.0037, 0.0039, and 0.0029, and standard deviations of 0.0038 and 0.0052, 0.0083 and 0.0021, and 0.0044 and 0.0020, at 10×, 20×, and 40×, respectively. In all cases, the standard deviations are bigger than the differences in averages, except one of the standard deviations at 20×, and another one at 40×. This is in contrast with the previous case of the ganglion, where the magnification at 10× for refractive index variance gives the best potential results. This fact demonstrates a dependence of discrimination potential on the tissue type, as expected.

The previous analysis of averages and standard deviations is of interest, but requires a deeper statistical analysis to evaluate the discrimination potentiality of the phase-contrast parameters. For instance, as shown previously, it was difficult to evaluate the potentiality when standard deviations are similar to the difference between the average values, or even when one standard deviation is bigger, while the other one is smaller. The ANOVA analysis of Table 2 tries to contribute to this analysis. Table 2 shows the *p*-values of each combination of tissue type, phase-contrast parameter, and magnification. A lower *p*-value indicates a bigger statistical difference in the average values of healthy and tumoral tissues. *p*-values below 0.005, that is usually taken as a reference for significant difference, are marked in bold on the table. As it can be seen on Table 2, each tissue type has at least two phase-contrast parameters that provide significance below 0.005 at particular magnifications. This fact reinforces the idea that these phase-contrast parameters are of interest for tissue discrimination of healthy and tumoral state of liver, kidney, ganglion, testicle, and brain, as previously shown for colon, kidney, and liver [28]. Refractive index variance and scattering coefficient present enough significance for all the considered tissue types. This significance changes according to magnification, but the refractive index variance at 10×, and the scattering coefficient at 20× are significant for all the tissues. These results are in agreement with the assumption that the spatial distribution of the refractive index could be a relevant discriminative property, as these two phase-contrast parameters are directly related with it. The refractive index variance is the direct measurement of the spatial refractive index distribution, and the scattering coefficient is strongly related with it, as the spatial heterogeneity gives rise to scattering. The fractal dimension is significant only for liver, ganglion, and brain, and the anisotropy factor and the outer scale provide potential significance for ganglion and brain only. Although the anisotropy factor is also a scattering property, it usually depends on the size of the inhomogeneities. The outer scale and the fractal dimension are also parameters related with the scale of the inhomogeneities. This relationship with the scale is more tissue type dependent, as the inner structures of each biological tissue present morphological and physiological differences, that could change more or less in a tumoral state. According to Table 2, all the phase-contrast parameters are significant, at least for some magnifications, for ganglion and brain. Refractive index variance, scattering coefficient, and fractal dimension are significant for the liver, and refractive index variance and scattering coefficient are of potential interest, at some magnifications, for kidney and testicle. Regarding magnifications, phase-parameters at 10× are significant for brain tissue, while there is no single magnification to make all parameters significant for ganglion. The three parameters of interest for liver can be measured at 20×. In the case of the kidney, the two potential parameters at 20× or 40× remain significant. Finally, testicle images at 40× can provide the two significant parameters. From the previous analysis it seems that tissue type heterogeneity makes it difficult to select particular phase-contrast parameters at particular magnifications. These results are in accordance with the previous ones reported for colon, kidney and liver [28]. As a consequence, all the phase-contrast parameters are considered for the automation discrimination problem, at all the magnifications considered.

Phase-contrast parameters of each tissue type are used to train and test eight different automatic classifiers. First, all the data are used to train each classifier, and then the classifiers are tested with the same data to check if they can provide a correct classification or not. This re-substitution error results appear in Table 3. The re-substitution error has been divided into the false positive (FP) and false negative (FN) rates. Total re-substitution errors, calculated as the sum of FP and FN, under 0.1 (or 10%) are marked in bold. A first look at Table 3 shows a great variability between the classifiers, as expected. Decision Tree (DT) classifier is the only one that presents errors below 10% for all the tissue types, for any of the magnifications. This error goes down to 0 for testicle tissue at 40×, with a maximum of 8.73% for liver at 10×. The classifier is highly specific for testicle and brain, even with 100% specificity at 10× for both tissues, and also at 20× for brain, and at 40× for testicle. DT is also quite sensitive for testicle at 20× and 40×, with 100% sensitivity. On the other hand, kNN and SVM classifiers are not able to provide a classification with errors below 10% for any of the possible combinations of tissue type and magnification, with a minimum of 12.5% for kNN on testicle at 10×, and 26.26% for SVM on kidney at 20×. kNB and ANN are able to provide errors below 10% for all the tissues but kidney. In the case of kNB this can be done at 20× for all the tissues, while for ANN there is no single magnification that provides the desired results for all the tissue types. The minimum error for kNB is 2.78% for testicle at 10× and 20×, and the minimum for ANN is 1.39% for testicle at 40×. The other classifiers, LDA, QDA, and NB are able to provide errors below 10% for three of the tissue types, liver, ganglion and testicle in the case of LDA, and ganglion, testicle and brain for QDA and NB. Minimum error for LDA is 2.78%, for QDA 1.39%, and for NB 6.95%, all of them for testicle at 40×. The results of Table 3 are presented graphically in Figure 3 to facilitate the analysis. The graphs in Figure 3 confirm that DT presents low re-substitution error values for all the tissue types, while SVM presents high values in general. Analyzing data on a tissue type basis, DT presents small error for brain as said, but QDA at 20× is also a good classifier. In the case of ganglion, DT, particularly at 10× and 20× is the best one. Liver and kidney also show that the best choice is DT at 20× for liver, and DT at 10× for kidney. In the case of testicle there are low errors with DT, QDA at 20× or 40×, and kNB at 10× or 20×. Prevalence of specificity or sensitivity is, as said before, dependent on the tissue type, classifier and magnification. From this analysis of re-substitution error, DT classifier would be applicable to all the tissue types with an error below 10% for any magnification. Although ANN would provide an error with the same characteristics for all the tissue types but kidney, in this case the error has a minimum of 22.68% at 20× and 40×, that is quite high. kNB provides classification errors below 10% for all but kidney tissue, in this case with a minimum of 16.28% at 40×.

Although the previous results are promising, it would be more realistic to employ a group of data for training the classifiers and the rest for testing. This gives rise to the cross-validation error, that also tries with several different groups of data to avoid bias when selecting the training and testing groups. Table 4 shows the results of the cross-validation errors for the eight previous classifiers. Again, the total cross-validation errors, obtained as a sum of FP and FN, below 0.1 (10%) are marked in bold. In general, the cross-validation errors are bigger than the re-substitution errors, as can be seen in Table 4, and was expected by definition. There is now no classifier able to provide errors below 10% for all the tissue types. ANN is able to provide this outcome for all tissue types but kidney. However, there is no common magnification factor that can assure these results, and at least 20× and 40× should be used. The minimum error is 1.43% for testicle at 40×, and a maximum of 5.72% for testicle at 10×. There is also a maximum specificity, 100%, for testicle at 40×. As expected, now that the errors increased, SVM and kNN are not yet capable to classify with errors less than 10% for any tissue type. The minimum error for SVM is 28.04% for kidney at 20×, and 16.78% for testicle at 10×. LDA can give an error below 10%, 2.68%, only for testicle at 40×, and even with a 100% specificity. kNB and NB give these results for only two tissues, ganglion and testicle, at 20× for both in the case of kNB and minimum of 3.93% for testicle at 20×, and with no common magnification for NB, and a minimum of 6.79% for testicle at 40×. DT and QDA can provide errors below 10% for three tissues, ganglion, testicle and brain, and a minimum of 5.72% for testicle at 40× for DT, and of 2.68% for testicle at 40× for QDA, with a specificity of 100%. Figure 4 shows the same values graphically for each of the tissue types. In the case of brain ANN at 40×, QDA at 20× and DT at 10× are the most appropriate classifiers. Ganglion tissue presents good results for ANN at 10× and 20×, and DT at 20×. Liver presents good results for ANN at 20×. Kidney is the most difficult tissue to classify, as there are no results below 10%, as said. The potential influence of the uneven distribution of images in the classification results is not clearly reflected in the results. Kidney and liver present the maximum number of images, with a total of 516 each, so the classification algorithms should work better. However, while for liver it is possible to find error results under 5% for ANN at 20×, it is not possible to find results better than 19% for kidney. Tissues with a lower number of images, such as ganglion with 324, testicle with 216 and brain with 162, present best results of 1.86% for DT at 20×, 1.39% for QDA at 40×, and 1.85% for QDA at 20×. There is also no apparent correlation of the best results with the minimum error given by the classification algorithms. Figure 4 establishes that ANN and DT are the best classifiers. According to Table 4, the minimum error is obtained from DT at 20×, 19.25%, while ANN has a minimum error of 19.64% at 40×. Lastly, testicle tissue has reduced errors for ANN at 20× and 40×, and LDA and QDA at 40×. These results show that a good potential classifier would be ANN with a combination of at least 20× and 40× magnifications. In this case, all the tissues but kidney have a maximum error of 4.68% for liver at 20×, and kidney could present a minimum error of 19.64% at 40×. An alternative would be DT, which was the best in the re-substitution error analysis, with tissues other than liver and kidney with a maximum error of 9.67% for brain at 10×, and capable of having errors of 13.36% for liver at 20×, and 19.25% for kidney at 20×. The case of QDA could be also an option, but in this case the minimum for kidney would be an error of 28% at 20×, that is quite high. 

## 5. Conclusions

The previous analysis has shown that the proposed phase-contrast parameters are potential candidates for tumoral tissue discrimination in an automatic artificial intelligence system based on phase-contrast microscopic images of five different fresh biological tissues. The use of phase-contrast parameters from microscopic images allows refractive index distribution information to be obtained without a strong dependence on particular pixel-wise data, as is the case when the information of each pixel of the whole image is employed. It also simplifies the classification algorithms training and implementation, and as a consequence, the feasibility of implementation and the speed of diagnosis increase. The present proposal employs fresh biological tissue slices, in which no fixation nor staining procedures were applied. The system provides a completely automated discrimination diagnosis, based on motorized microscopic images acquisition and the application of a trained classifier. The use of label-free samples could be of great interest in clinical practice for time to diagnosis reduction, artifacts avoidance, and digital diagnosis. This could be particularly critical when a quick diagnosis is needed, such as during surgical interventions of tumoral tissue removal. In this case the samples can be sliced and directly located on the system for automated diagnosis. The present system is more affordable than quantitative phase imaging approaches that would facilitate adoption at histopathology services in hospitals. The cost of the system could be even compensated by the elimination of reactants and reduction of processing steps, specially for low-cost devices in developing countries.

The analysis of averages and standard deviations and the ANOVA results, indicate that there is a tissue type and magnification dependence on the statistical significance of the differences between healthy and tumoral tissues. This dependence does permit clearly selecting particular phase-contrast parameters or a particular magnification for a general multi-tissue device. The results of the application of different classifiers show that it is possible to obtain an automatic classifier based on ANN with a maximum cross-validation error of 4.68% for liver, ganglion, testicle, and brain, and 19.64% for kidney. Although the last error could be too high, it just affects one of the tissue types, while the others remain under 5%. An alternative would a DT classifier, with errors below 10% for ganglion, testicle, and brain, 13.36% for liver, and 19.25% for kidney. The last error could still be too high, but again it appears on only one tissue type. Further studies can employ other classification algorithms to try to improve the results for kidney, or even more advanced phase-contrast imaging systems. Although a quite broad group of tissue types is being used in this work, additional tissue types could be also tested. In any case, the results show the feasibility of considering refractive index distribution as a tumoral discriminant on label-free biological samples.

## Figures and Tables

**Table 1:** Results of average and standard deviation of each of the phase-contrast parameters for the tissue type, state and magnification shown.

Sample ^1^	RIV ^2^	SC ^3^ [rad^2^/mm]	AF ^4^	FD ^5^	OS ^6^ [µm]
10x liver H	0.0244 ± 0.0059	18.15 ± 20.30	0.98087 ± 0.05989	2.707 ± 0.250	81.56 ± 8.65
10x liver T	0.0267 ± 0.0041	32.17 ± 30.34	0.88799 ± 0.52312	2.525 ± 0.363	80.18 ± 14.84
20x liver H	0.0217 ± 0.0049	36.57 ± 29.09	0.99653 ± 0.00472	2.670 ± 0.285	40.90 ± 3.45
20x liver T	0.0169 ± 0.0080	20.81 ± 19.99	0.99802 ± 0.00324	2.485 ± 0.405	39.10 ± 5.57
40x liver H	0.0124 ± 0.0050	23.29 ± 14.78	0.99932 ± 0.00030	3.194 ± 0.207	25.40 ± 2.54
40x liver T	0.0128 ± 0.0067	25.99 ± 26.17	0.99924 ± 0.00038	3.129 ± 0.309	24.60 ± 3.77
10x kidney H	0.0278 ± 0.0058	25.77 ± 26.86	0.95906 ± 0.14489	2.402 ± 0.357	81.18 ± 26.20
10x kidney T	0.0246 ± 0.0065	19.53 ± 25.48	0.99178 ± 0.01074	2.529 ± 0.372	81.32 ± 13.49
20x kidney H	0.0168 ± 0.0051	23.94 ± 17.52	0.99856 ± 0.00147	2.553 ± 0.487	41.38 ± 7.87
20x kidney T	0.0194 ± 0.0040	41.66 ± 19.09	0.99897 ± 0.00083	2.647 ± 0.384	41.44 ± 6.16
40x kidney H	0.0104 ± 0.0041	14.50 ± 16.04	0.99871 ± 0.00252	3.215 ± 0.273	26.69 ± 4.87
40x kidney T	0.0136 ± 0.0037	30.45 ± 16.89	0.99937 ± 0.00030	3.172 ± 0.320	25.67 ± 4.62
10x ganglion H	0.0223 ± 0.0042	11.10 ± 15.23	0.99132 ± 0.00988	2.615 ± 0.163	78.76 ± 4.83
10x ganglion T	0.0290 ± 0.0043	16.37 ± 26.96	0.96354 ± 0.04785	2.218 ± 0.255	71.32 ± 5.31
20x ganglion H	0.0206 ± 0.0021	43.46 ± 12.91	0.99913 ± 0.00017	2.629 ± 0.132	39.54 ± 2.45
20x ganglion T	0.0144 ± 0.0063	18.07 ± 15.31	0.99884 ± 0.00036	2.665 ± 0.416	41.05 ± 6.36
40x ganglion H	0.0108 ± 0.0042	18.39 ± 11.12	0.99938 ± 0.00013	3.275 ± 0.233	26.26 ± 4.62
40x ganglion T	0.0126 ± 0.0035	22.74 ± 16.10	0.99930 ± 0.00020	3.166 ± 0.222	24.51 ± 2.75
10x testicle H	0.0201 ± 0.0038	21.76 ± 24.11	0.93896 ± 0.11773	2.661 ± 0.254	80.36 ± 7.43
10x testicle T	0.0238 ± 0.0052	33.17 ± 37.61	0.98946 ± 0.02517	2.558 ± 0.311	78.10 ± 7.05
20x testicle H	0.0143 ± 0.0083	16.99 ± 15.30	0.99882 ± 0.00077	2.777 ± 0.699	45.26 ± 11.38
20x testicle T	0.0182 ± 0.0021	39.35 ± 21.37	0.99888 ± 0.00178	2.617 ± 0.242	39.61 ± 3.43
40x testicle H	0.0156 ± 0.0044	39.12 ± 17.51	0.99932 ± 0.00043	3.123 ± 0.547	26.16 ± 6.65
40x testicle T	0.0127 ± 0.0020	27.96 ± 13.16	0.99943 ± 0.00020	3.144 ± 0.207	24.20 ± 2.52
10x brain H	0.0261 ± 0.0019	66.19 ± 24.08	0.99693 ± 0.00677	2.428 ± 0.065	74.67 ± 3.57
10x brain T	0.0302 ± 0.0032	43.64 ± 35.87	0.99349 ± 0.00748	2.179 ± 0.155	70.42 ± 3.60
20x brain H	0.0148 ± 0.0013	17.18 ± 6.24	0.99886 ± 0.00019	2.604 ± 0.065	39.16 ± 1.47
20x brain T	0.0138 ± 0.0048	15.97 ± 8.98	0.99891 ± 0.00028	2.683 ± 0.373	40.87 ± 4.96
40x brain H	0.0097 ± 0.0010	10.60 ± 2.94	0.99923 ± 0.00007	3.249 ± 0.075	25.08 ± 1.42
40x brain T	0.0099 ± 0.0031	16.03 ± 12.24	0.99940 ± 0.00015	3.322 ± 0.194	26.38 ± 2.83

^1^ H = healthy, T = tumoral; ^2^ RIV = Refractive Index Variance; ^3^ SC = Scattering Coefficient; ^4^ AF = Anisotropy Factor; ^5^ FD = Fractal Dimension; ^6^ OS = Outer Scale.

**Table 2:** Results of the ANOVA analysis for each tissue type, magnification and phase-contrast parameter. The *p*-value of the test is shown. *p*-values below 0.005 are marked in bold.

Parameter	Magnification	Liver	Kidney	Ganglion	Testicle	Brain
	10x	**0.0040**	**8.0806·10^−4^**	**1.4369·10^−14^**	**0.0011**	**5.9980·10^−15^**
**RIV** ^1^	20x	**4.4433·10^−6^**	**3.1213·10^−4^**	**0.0032**	0.0085	**6.5141·10^−9^**
	40x	0.5945	**1.9982·10^−7^**	0.0529	**6.5115·10^−4^**	**1.3054·10^−4^**
	10x	**4.8004·10^−4^**	0.1197	**0.0019**	0.1300	**1.0044·10^−11^**
**SC**^2^ [rad^2^/mm]	20x	**5.4952·10^−5^**	**1.9922·10^−9^**	**1.4394·10^−5^**	**2.7525·10^−6^**	**0.0013**
	40x	0.4064	**1.9241·10^−9^**	0.2551	**0.0032**	**0.0035**
	10x	0.1037	0.0383	0.3281	0.0141	**2.8297·10^−4^**
**AF** ^3^	20x	0.0164	0.0242	**5.3934·10^−6^**	0.8691	0.1726
	40x	0.1730	0.0168	**0.0047**	0.1625	0.0230
	10x	0.0578	0.0235	**1.1789·10^−19^**	0.1273	**1.9627·10^−14^**
**FD** ^4^	20x	**7.0542·10^−4^**	0.1613	0.2120	0.2003	**3.4772·10^−6^**
	40x	0.1081	0.3510	0.0074	0.8295	**0.0019**
	10x	0.4571	0.9641	**1.0577·10^−12^**	0.1898	**6.8376·10^−7^**
**OS**^5^ [µm]	20x	0.0120	0.9517	0.8872	0.0058	**1.8737·10^−4^**
	40x	0.1068	0.1628	0.0092	0.1041	0.0286

^1^ RIV = Refractive Index Variance; ^2^ SC = Scattering Coefficient; ^3^ AF = Anisotropy Factor; ^4^ FD = Fractal Dimension; ^5^ OS = Outer Scale.

**Table 3:** Results of the re-substitution error, expressed separately for the false positive and false negative rates, for each tissue type and magnification. Combinations with total re-substitution error (FP + FN) < 0.1 are marked in bold.

Classifier	Error Rate ^1^	Magnification	Liver	Kidney	Ganglion	Testicle	Brain
**LDA** ^2^	FP	10x	0.1462	0.1570	0.0463	0.0833	0.0741
		20x	**0.0349**	0.1520	**0.0463**	**0.0417**	0.3333
		40x	0.1124	0.1047	0.2358	**0**	0.0741
	FN	10x	0.2047	0.2267	0.0556	0.0278	0.0370
		20x	**0.0640**	0.1345	**0.0370**	**0.0556**	0
		40x	0.2249	0.1453	0.0755	**0.0278**	0.0556
**QDA** ^3^	FP	10x	0.0351	0.4012	**0.0370**	0.1250	**0**
		20x	0.0523	0.1813	**0.0278**	**0**	**0**
		40x	0.0592	0.3372	0.0660	0	0.0185
	FN	10x	0.3801	0.0349	**0.0370**	0.0139	**0.0556**
		20x	0.0523	0.0585	**0.0093**	**0.0278**	**0.0185**
		40x	0.3018	0.0174	0.2075	**0.0139**	0.0926
**kNB** ^4^	FP	10x	0.1279	0.0930	**0.0278**	**0.0139**	0
		20x	**0.0407**	0.1105	**0.0278**	**0.0278**	**0**
		40x	0.1279	0.0640	0.0648	**0.0417**	0.0185
	FN	10x	0.1105	0.0872	**0.0556**	**0.0139**	0.1667
		20x	**0.0291**	0.0930	**0.0370**	**0**	**0.0926**
		40x	0.1395	0.0988	0.1204	**0**	0.1111
**NB** ^5^	FP	10x	0.0407	0.3953	**0.0648**	0.2500	0.0185
		20x	0.0523	0.1395	0.0556	**0.0694**	**0.0185**
		40x	0.0988	0.3198	0.2593	**0.0139**	0.0556
	FN	10x	0.3837	0.0349	**0.0278**	0.1806	0.1296
		20x	0.0581	0.1221	0.0648	**0.0278**	**0.0741**
		40x	0.2616	0.0174	0.1389	**0.0556**	0.0741
**kNN** ^6^	FP	10x	0.0988	0.0814	0.1759	0.0694	0.0556
		20x	0.1163	0.1105	0.0926	0.0694	0.1296
		40x	0.1221	0.0988	0.2037	0.1528	0.1481
	FN	10x	0.1221	0.1453	0.0833	0.0556	0.0741
		20x	0.1395	0.1105	0.0648	0.0972	0.0741
		40x	0.1453	0.0640	0.0648	0.1389	0.0741
**SVM** ^7^	FP	10x	0.3953	0.3314	0.3148	0.1944	0.2963
		20x	0.2326	0.1395	0	0.4583	0.2407
		40x	0.0233	0.0349	0.0741	0.0417	0
	FN	10x	0.2151	0.0872	0.0926	0.3194	0.3333
		20x	0.1221	0.1221	0.6667	0.2639	0.2407
		40x	0.4360	0.3430	0.4630	0.4861	0.6481
**DT** ^8^	FP	10x	**0.0640**	**0.0349**	**0.0093**	**0**	**0**
		20x	**0.0174**	**0.0523**	**0.0093**	**0.0139**	**0**
		40x	**0.0116**	**0.0465**	**0.0463**	**0**	**0.0370**
	FN	10x	**0.0233**	**0.0233**	**0.0093**	**0.0139**	**0.0370**
		20x	**0.0116**	**0.0233**	**0.0093**	**0**	**0.0741**
		40x	**0.0640**	**0.0233**	**0.0463**	**0**	**0.0185**
**ANN** ^9^	FP	10x	0.2907	0.1395	**0.0278**	**0.0417**	0.0556
		20x	**0.0349**	0.1163	0.1111	**0.0417**	**0.0370**
		40x	0.0988	0.1105	0.0926	**0.0139**	0
	FN	10x	0.0814	0.4302	**0.0093**	**0.0139**	0.1481
		20x	**0.0291**	0.1105	0.1019	**0.0417**	**0.0556**
		40x	0.1512	0.1163	0.1667	**0**	0.3333

^1^ FP = False Positive rate; FN = False Negative rate; ^2^ LDA = Linear Discriminant Analysis; ^3^ QDA = Quadratic Discriminant Analysis; ^4^ kNB = kernel Naïve-Bayes; ^5^ NB = Naïve-Bayes; ^6^ kNN = k-Nearest Neighbors; ^7^ SVM = Support Vector Machine; ^8^ DT = Decision Tree; ^9^ ANN = Artificial Neuronal Network.

**Table 4:** Results of the cross-validation error, expressed separately for the false positive and false negative rates, for each tissue type and magnification. Combinations with total cross-validation error (FP + FN) < 0.1 are marked in bold.

Classifier	Error Rate ^1^	Magnification	Liver	Kidney	Ganglion	Testicle	Brain
**LDA** ^2^	FP	10x	0.1510	0.1807	0.0545	0.1089	0.0867
		20x	0.0408	0.1454	0.0555	0.0411	0.3167
		40x	0.1222	0.1157	0.2682	**0**	0.1333
	FN	10x	0.2157	0.2373	0.0564	0.0536	0.0767
		20x	0.0814	0.1513	0.0464	0.0696	0.0167
		40x	0.2614	0.1503	0.0655	**0.0268**	0.0700
**QDA** ^3^	FP	10x	0.0526	0.4072	0.0636	0.1679	0.0600
		20x	0.0585	0.2039	**0.0464**	**0.0268**	**0.0767**
		40x	0.1046	0.3670	0.1473	**0**	0.0967
	FN	10x	0.3778	0.0350	0.0382	0.0268	0.0767
		20x	0.0578	0.0761	**0.0191**	**0.0429**	**0.0200**
		40x	0.3196	0.0288	0.2218	**0.0268**	0.1300
**kNB** ^4^	FP	10x	0.1856	0.1343	0.0545	0.1536	0.0600
		20x	0.0408	0.1458	**0.0464**	**0.0125**	0.0367
		40x	0.1392	0.1219	0.1200	**0.0429**	0.0567
	FN	10x	0.1510	0.1680	0.0564	0.0411	0.1833
		20x	0.0641	0.1333	**0.0373**	**0.0268**	0.0733
		40x	0.2206	0.1039	0.1600	**0.0536**	0.1267
**NB** ^5^	FP	10x	0.0467	0.4013	**0.0636**	0.2946	0.0400
		20x	0.0523	0.1454	0.0564	**0.0411**	0.0767
		40x	0.0984	0.3088	0.2500	**0.0143**	0.0767
	FN	10x	0.3716	0.0405	**0.0291**	0.1375	0.1367
		20x	0.0637	0.1461	0.0555	**0.0554**	0.0733
		40x	0.2732	0.0288	0.1482	**0.0536**	0.1233
**kNN** ^6^	FP	10x	0.1516	0.1575	0.2573	0.0839	0.1100
		20x	0.1866	0.1513	0.1127	0.1518	0.1233
		40x	0.2150	0.1333	0.2773	0.2089	0.1667
	FN	10x	0.1582	0.1680	0.1664	0.0839	0.0967
		20x	0.1575	0.1503	0.1109	0.1393	0.0567
		40x	0.2141	0.0931	0.1418	0.1929	0.1300
**SVM** ^7^	FP	10x	0.2435	0.1856	0.3145	0.2786	0.2533
		20x	0.1353	0.0990	0.0364	0.3625	0.2433
		40x	0.2428	0.0706	0.0927	0.0982	0.0333
	FN	10x	0.2621	0.3546	0.0918	0.1946	0.2967
		20x	0.3765	0.1814	0.6309	0.2518	0.2167
		40x	0.2461	0.3954	0.3882	0.3179	0.6000
**DT** ^8^	FP	10x	0.1503	0.0987	0.0455	0.0696	**0.0200**
		20x	0.0699	0.0824	**0.0373**	**0.0268**	0.0533
		40x	0.0935	0.1222	0.1473	**0.0143**	0.0967
	FN	10x	0.1160	0.1111	0.0636	0.0679	**0.0767**
		20x	0.0637	0.1101	**0.0282**	**0.0554**	0.1233
		40x	0.1389	0.0980	0.1009	**0.0429**	0.1233
**ANN** ^9^	FP	10x	0.1216	0.0925	**0.0182**	**0.0429**	0.0533
		20x	**0.0118**	0.1163	**0.0182**	**0.0143**	0.0533
		40x	0.1333	0.1042	0.1482	**0**	**0.0333**
	FN	10x	0.1451	0.1458	**0.0191**	**0.0143**	0.0567
		20x	**0.0350**	0.1052	**0.0191**	**0.0143**	0.0700
		40x	0.0866	0.0922	0.0736	**0.0143**	**0.0167**

^1^ FP = False Positive rate; FN = False Negative rate; ^2^ LDA = Linear Discriminant Analysis; ^3^ QDA = Quadratic Discriminant Analysis; ^4^ kNB = kernel Naïve-Bayes; ^5^ NB = Naïve-Bayes; ^6^ kNN = k-Nearest Neighbors; ^7^ SVM = Support Vector Machine; ^8^ DT = Decision Tree; ^9^ ANN = Artificial Neuronal Network.

## Data Availability

The data presented in this study are available on request from the corresponding author. The data are not publicly available due to privacy.
